# Psychiatrists’ Experience of Vocational Rehabilitation for Patients with Mental Illness

**DOI:** 10.1007/s11126-021-09896-w

**Published:** 2021-03-04

**Authors:** Åsa Wallentin, Emelie Kinberg, Jennifer Strand, Peter Sand

**Affiliations:** 1 Department of Psychiatry, Sahlgrenska University Hospital, Gothenburg, Sweden; 2 Department of Psychology, Gothenburg University, Gothenburg, Sweden; 3 Department of Psychiatry and Neurochemistry, Institute of Neuroscience and Physiology, The Sahlgrenska Academy, Sahlgrenska University Hospital, University of Gothenburg, Gothenburg, Sweden

**Keywords:** Psychiatry, Psychiatrists, Mental illness, Vocational rehabilitation, Qualitative study

## Abstract

The aim of this study was to explore psychiatrists’ experience of vocational rehabilitation for patients with mental illness. The study employed a qualitative design to explore psychiatrists’ experience of vocational rehabilitation. Ten psychiatrists, five women and five men, 33–62 years of age (median 40), were interviewed. All of them worked with patients at Sahlgrenska University Hospital. The interviews were analyzed using inductive thematic analysis. All participants considered vocational rehabilitation to be of great importance for patients’ well-being and health. The results were characterized by two opposite experiences: frustration and agency, these were the two main themes in the analysis. All narratives embraced both experiences, but some reflected more frustration and others more agency. In order for the psychiatrist to master the assignment, there is a need for further training and supervision. The psychiatrist’s role, as well as other professional roles within the team, requires clarification, and the support from rehabilitation coordinators and occupational therapists should be enhanced. There is a need for improved cooperation with external actors.

## Introduction

Mental illness often lead to recurrent episodes of impaired work ability, which incurs high costs for both the affected individual and the wider society [1, 2]. Vocational rehabilitation is a multidisciplinary effort to support a person with an illness or disability to access, maintain or return to the labour market or other meaningful occupation [3, 4].

In Sweden, the conditions for physicians and other actors working with vocational rehabilitation have gone through substantial changes [5]. Around the year 2000 there was a peak in sick leave, which sparked public debate and reform of the social insurance system [5]. One of the main changes was the Social Insurance Agency (SIA) taking on the role of gatekeeper of resources by requiring clear assessments for sick leave and performing more rigorous control of sickness certificates [5]. Other examples of important reforms were to include the topic of insurance medicine, the medical assessment of work disability for insurance purposes, in the training of resident physicians (starting in 2015) and to mandate the National Board of Health and Welfare to supervise and issue guidelines for sickness certification [5]. There has also been an expansion of research exploring evidence-based methods of helping people with severe mental disorders to gain employment; this has resulted in higher levels of employment [6–9], improved quality of life and improvements in mental health [7, 9, 10].

Vocational rehabilitation is a shared responsibility including stepwise interventions and assessments from various professionals in the mental health services, the municipality, the Social Insurance Agency (SIA) and the Public Employment Service (PES). The SIA is responsible for the coordination of these different interventions [11].

The physician’s responsibility in vocational rehabilitation is to determine the disease or disability and its effect on the patient’s functioning and work ability, discuss advantages and disadvantages of sick leave with the patient, determine the degree and duration of sick leave, consider alternative interventions, provide a sickness certificate if applicable and collaborate with other actors [12]. Actors within the health care system are, for example, rehabilitation coordinators, occupational therapists, physiotherapists, psychologists and nurses [12]. Rehabilitation coordinators constitute a relatively new professional group which has gradually been implemented in Swedish health care to facilitate internal coordination and collaboration with external actors regarding vocational rehabilitation [5]. About a quarter of psychiatrists have access to rehabilitation coordinators [5]. The physician also collaborates with external actors, such as the administrators at the SIA and the PES, and they all have to follow relevant legislation [4]. For example, according to the Swedish social security law, the work ability of a person needs to be reduced by 25%, 50%, 75% or 100% to qualify for sick pay [13]. This law is especially relevant to this study, since the gap between no work ability and 25% sometimes requires the physician to plan for pre-vocational training. The physician also collaborates with local coordination associations, which develop rehabilitation interventions to help individuals return to work; these associations are co-financed by the SIA, PES, healthcare authority and municipality [14].

In vocational rehabilitation, the physician takes on two sometimes conflicting roles: being both the treating physician and the medical expert [12]. According to the first role, the physician’s aim is to understand, create trust and offer help, while the aim of the second role is to provide objective information about the patient to other authorities [12]. Almost half of the psychiatrists in Sweden report that managing the two roles is very or fairly problematic [15]. A majority of the psychiatrists experience, at least once a week, lack of time to work with vocational rehabilitation, and a majority report that it is very or fairly problematic to assess work capacity and give a long-term prognosis [15].

There are no qualitative studies on psychiatrists’ experiences of working with vocational rehabilitation. Most of the Swedish qualitative studies have been performed in primary care settings. In this clinical context, physicians have described a lack of knowledge of the social security system, the patient’s workplace and the labour market in general [16]. Physicians have also expressed frustration with sickness certification and the use of unsanctioned techniques to convince the SIA of the patient’s need for sick leave [17]. Physicians have described collaboration with external actors as important but scarce and difficult to achieve [18]. Other actors describe problems in getting in touch with physicians [16] and physicians have described difficulties in getting in touch with the SIA [18, 19]. Physicians have also reported that other internal actors are rarely active in the process and that there is a lack of structure for collaboration [18].

A study on psychiatrists’ experience of working with vocational rehabilitation for patients with mental illness is motivated for several reasons. According to the regional medical guidelines, psychiatry is supposed to treat mental health patients with complex symptoms and low function, or patients who have received treatment in primary care which has not led to improvements [20]. Furthermore, patients who are treated for mental illness have longer periods of sick leave and often relapse after rehabilitation efforts are made [21–24]. These factors could have an effect on the psychiatrists’ experience of working with vocational rehabilitation. Consequently, the aim of this study is to explore psychiatrists’ experience of vocational rehabilitation for patients with mental illness.

## Materials and Methods

### Design

The study employed a qualitative design to explore the meaning and significance of psychiatrists’ experience of vocational rehabilitation through interviews at Swedish clinics for patients with mental illness. The study design was reviewed and approved by the Swedish Ethical Review Board (approval number: 2019/978–18).

### Recruitment and Sampling

All psychiatrists (N = 10) at two outpatient clinics were asked to participate in the study. They received information at a physical meeting or by telephone. Information included the aim of the study, the voluntary nature of participation and a statement that confidentiality would be assured. This generated five participants. Then five more participants were strategically sampled from the psychiatrists (N = 12) working at two other outpatient clinics to maximize data variation. Strategic sampling was performed according to variation in sex, age, professional qualification (Swedish or foreign) and length of work experience as a psychiatrist. Strategic sampling was performed with help of the managers of the outpatient clinics, who mediated contact between the researchers and the psychiatrists. Two sampled informants who were asked to participate declined. All participants signed a written consent form before data collection.

Ten physicians with a specialist qualification in psychiatry, aged between 33 and 62 (median 40) years, five men and five women, from four outpatient clinics were recruited to the study. These clinics treated patients with mental illness such as depression, anxiety, personality disorders, PTSD, OCD, Bipolar Disorders (without Type 1 Disorder) and neuropsychiatric conditions. The clinics, in different geographic areas of a large Swedish city, were organizationally part of the department of psychiatry for patients with mental illness at Sahlgrenska University Hospital. The participants had between 4 and 21 (median 9) years of experience as specialists in psychiatry. Three had a Swedish medical degree, and seven had a medical degree from another European country. Five had completed their training as resident physicians in Sweden, five of them abroad.

### Data Collection

The interviews were performed in the participant’s office. The interviews lasted between 34 and 72 min. The first author conducted six interviews and the second author conducted four. The interview guide was semi-structured and covered three areas: the psychiatrist’s experience of vocational rehabilitation, perceived problematic and helpful factors in collaboration, and how the participant would prefer the vocational rehabilitation process to be organized. Participants were asked to talk about vocational rehabilitation interventions, except those which are strictly pharmacological or psychotherapeutic. All questions were open-ended to invite the psychiatrists’ own thoughts and experiences. Follow-up questions were used to a varying extent, depending on how much detail the participant supplied spontaneously. All interviews were audio-recorded and transcribed verbatim.

### Data Analysis

The interviews were analyzed using inductive thematic analysis, which is described as a flexible qualitative method of analysis where patterns of meaning in data are reported through a rich and detailed description of themes [25]. The analysis was performed using Braun and Clarke’s [25] six-phase guide for thematic analysis.

In the first phase, the authors familiarized themselves with the data by reading the transcribed interviews and making notes, generating an initial list of ideas that were interesting and were relevant to the aim. In the second phase, the first author labelled short extracts of the psychiatrists’ statements with descriptive codes according to their content, semantic or latent. The coding was performed without trying to fit the material into any pre-existing framework and continued until the content of the entire data set was coded. In the third phase, the codes were sorted into potential themes. This process generated an initial mind map of themes and sub-themes. The themes and sub-themes were then discussed within the research group, considering how each theme contributed to the overall narrative of the material in relation to the research questions. New ideas that emerged were then used by the first author for recoding the material, generating a new mind map that was discussed in the research group. This process was repeated twice until the mind map was refined and a candidate thematic map had been created. The codes were then sorted into two overarching themes reflecting either the sense of agency or frustration in the process of vocational rehabilitation. In phase four, the themes were reviewed by the first author, reading all the coded data extracts for each theme to ensure that a coherent pattern was formed within each theme. After that, the entire data set was re-read to ensure that the themes accurately represented the data. In phase five, the essence of each theme was further defined and refined by the researchers, also further determining which detailed aspects of the data the theme captured. Themes and sub-themes were described in detail and the themes were given names. As the sixth and final phase, the report was produced. Direct quotes were used in the report to allow readers to evaluate the analysis. During the process, Braun and Clarke’s [25] 15-point-criteria for conducting ‘good’ thematic analysis were used.

## Results

All participants considered traditional vocational rehabilitation (pre-rehabilitation efforts, internships and then trying to find a suitable employer) to be of great importance for patients’ well-being and health. But the narratives were characterized by two opposite experiences in relation to vocational rehabilitation: *frustration* and *agency*. All narratives embraced both experiences, but some were more characterized by frustration, others more on agency. These experiences form the two main themes in the analysis. They can be read as two in-depth descriptions of experienced problematic factors and facilitating factors in relation to vocational rehabilitation.

### Frustration

The overarching theme of frustration brings into focus an inner experience characterized by feeling responsible but not being able to help the patients. The experience of frustration is outlined in the four sub-themes *Lack of competence*, *Lack of support*, *Lack of power* and *Questioning of the psychiatrist’s role*.

#### Lack of Competence

Participants described the procedures of vocational rehabilitation as unclear and difficult to overview. Some participants wished for increased clarity regarding general routines of how to work with vocational rehabilitation at the outpatient clinic, including concrete instructions. Furthermore, training regarding the social insurance system was described as insufficient or inappropriate, not generating the necessary understanding of the processes of vocational rehabilitation.

I think we should get better training, at least about insurance medicine. We should know more about it, and how it works, and what is most relevant. We get it in some way but not in such detail that we would like to have. (Participant 10).

Uncertainty about current research in the field of vocational rehabilitation was expressed by most participants, and some described frustration arising from a lack of applicable research in the area: “Regarding returning to work, research is practically non-existent” (Participant 1). Failure to follow the formal descriptions of sickness certification was related to this frustration.

In the following example, the participant describes how he renews the sickness certificate even if the assessment is that the patient has a work ability, a procedure that is not in line with SIA guidelines.

So I think, for example, that the patient could work in some kind of adapted job. (…) But the patient himself doesn’t want to and has been on sick leave for 10 years. Other doctors have met him and I just renew the sickness certificate. (Participant 8).

Lack of competence was associated with being unfamiliar with the Swedish social insurance system and the Swedish organization of interventions in vocational rehabilitation. This uncertainty was particularly regarded as an obstacle by participants having either an overseas education or having done the training as a resident physician in another country. This was also the case for participants who were new at the clinic. “Because I am new here in Sweden, I came here four years ago, so I do not know the system very well.” (Participant 8).

#### Lack of Power

Feeling a lack of power in the process of vocational rehabilitation was related to feelings of frustration and anger. One aspect of this experienced powerlessness was when external actors made decisions that differed from one patient to another, or had a rapid turnover of staff dealing with the patient, thus making their decisions arbitrary and difficult to influence.

During a period of one calendar year, I would suspect that the patient has at least two to four changes of administrator handling the patient’s case which means an incredible uncertainty on both sides. (Participant 3).

The participants stressed that collaboration with the SIA and PES was crucial to the process. Some had the impression that staff at the authorities seemed to lack time to communicate or meet when it was needed. As a result, the participants felt less able to influence the process. “There are such difficult working conditions at the SIA that they have not been able to attend as many coordination meetings as they did before.” (Participant 5).

Problems in the communication between the SIA and PES was also described as destructive to the process. When they could not agree on financing, or on a suitable intervention for a patient, the process could get stuck. One explanation for the difficulties in collaboration was differences in language traditions.

So it is also that, with the SIA, you speak ‘disability language’. And for rehabilitation, you speak ‘habilitation language’. For example, the Employment Office as an actor is very well aware of that dilemma: Should one describe the sickness or should one promote the healthy side? But it is precisely that, that even related actors in the social security system no longer speak the same language. (Participant 3).

Participants stated that the demand from the SIA for specific formulations concerning objective evidence in the written sickness certificates was problematic, since it led to a devaluation of the role of the psychiatrist as a medical expert.

As a doctor you stretch the boundaries more and more to declare dysfunction because … how should I put it … a more reasonable or moderate degree of dysfunction is not accepted at all as objective evidence of some degree of reduction in work ability. (Participant 3).

Lack of power was also experienced when the SIA or PES rules were perceived as obstacles. For example, the rule stating that the individual plan must include work training increasing from 25 to 100% over a year was considered unrealistic for patients who had been on sick leave for many years. Furthermore, the rule that the patient must have 25% work ability to be granted work-oriented interventions from the SIA or PES was considered a problem. Patients with less than 25% work ability were sent instead to, for example, general activity centres run by the municipality, which had no particular focus on supporting vocational rehabilitation. Frequent changes of the rules that governed the decision-making of SIA and PES administrators were also described as a problem.

Now the SIA is changing rules all the time, but something that has been bothersome (…) I don't know if they have new rules, but for a while they demanded that we should have very precise planning in connection with return to work, that you would know exactly when the patient could go back to which degree of work. (Participant 5).

The general lack of jobs suitable for this vulnerable group of people was also regarded as problematic. Participants described that the patients tended to be unattractive on the labour market because of their fragility and need for adjustments in work demands. “In this society there is a problem for our group of patients, that we don’t have many workplaces of that kind.” (Participant 1).

#### Questioning the Psychiatrist’s Role

Frustrated participants tended to question their role in the process. This included questioning the fact that both psychiatrist and primary physician were responsible for sickness certification. Being a medical expert in social insurance decisions and at the same time being responsible for the clinical treatment of the patient was described as a challenge by some participants. The two roles were sometimes found to be in conflict, for example when the participant assessed that the patient had a level of functioning sufficient to be able to work and the patient did not agree.

As a doctor, you have two different roles, you must be on the patient’s side, but at the same time, you should be a person in authority and be able to act in the process as a medical expert. It can sometimes be a little hostile … or it complicates the relationship with the patient a good deal. (Participant 6).

Participants also felt burdened by being responsible for an assessment that impacts the patient’s economic situation. Other expressed the wish that other social authorities would take more responsibility for vocational rehabilitation. “I would like to have another authority, responsible for vocational rehabilitation (…), motivational dialogs, offering adapted employment, physicians, sickness certification, everything.” (Participant 8).

### Agency

The experience of agency in the process of vocational rehabilitation is described in the sub-themes *Orientation*, *Having adequate support* and *Mastery of procedures*.

#### Orientation

Among participants who described themselves as active and competent in the process of vocational rehabilitation, narratives were characterized by feeling oriented in the process and their role. Some participants likened their sense of orientation to having a mental map of the process.

There is a need for some kind of orientation map, the one that I have drawn here (…) I have come up with it myself and it is just my way of being able to orientate myself among all these different interventions, authorities and actors. (Participant 6).

Being sufficiently oriented in the process of vocational rehabilitation was described as a necessary prerequisite for agency, enabling progression through the different steps of the process.

You identify what level in the process we are at and chart what we would need to do more to move forward in the process. So that structure could be imagined, because in some way you have a map of it, in different steps, where you try to place yourself together with the patient. (Participant 1).

Learning from experienced colleagues, working with rehabilitation, reading scientific articles and searching for information on the internet were described as different ways to gather information that helped them to acquire agency. “The occupational therapist was a driving force (…) She also – about research and what is going on – sent us information and so on, and there we had many good discussions.” (Participant 9).

Training from the SIA in writing sickness certificates, together with templates used at the clinic, were described as helpful. “I got training from the SIA, who taught me how to write a [sickness] certificate.” (Participant 4).

#### Adequate Support

Among participants describing a sense of agency, the experience of having access to adequate internal and external resources was prominent. Psychiatrists who reported receiving sufficient support from other professionals at the outpatient clinic during the rehabilitation process, commented that this enabled their sense of agency. All participants described collaboration with a rehabilitation coordinator at the outpatient clinic as valuable. Rehabilitation coordinators were described as supportive in making assessments, planning interventions and handling collaboration with external actors.

She can meet the patient herself (…) and then she usually does it in a really thorough way or delves deep into the case in some way. (Participant 2).

For the psychiatrist to experience a sense of agency, he or she had to feel supported by the outpatient team and feel that vocational rehabilitation was identified as an important issue also by the team.

Then you have to have it as a theme at the team conference (…) you also have to talk work-oriented rehabilitation, what kind of employment does the person have, is it thought that they should be able to start working, for when you start there, then you start to become more conscious in each treatment intervention (Participant 1).

For the psychiatrist to experience agency there also had to be well-functioning cooperation with external actors. When forming a plan for rehabilitation, continuity at the SIA and optional interventions on their part were emphasized as important. Otherwise the participants perceived it as impossible to plan and make a prognosis for the patient’s return to work. To build collaboration with a genuinely committed and independent administrator at the SIA was described as an integral part of a successful vocational rehabilitation process. *“*I think personal continuity is very important, genuine commitment.*”* (Participant 6).

The ability to collaborate and communicate with officers at other authorities was needed to modify the planned interventions or steps in the process. “I suppose my feeling is that once we have meetings and the SIA is there, things get easier.” (Participant 2).

Participants mentioned interventions provided by coordination associations organized by municipalities with the aim of bridging gaps between SIA, PES, social services and health care, often targeting individuals far from the labour market, as particularly helpful.

Another thing that has helped a lot is when there is information and ongoing projects to help patients getting into projects that support young people on sick leave to join the job market. (Participant 2).

#### Mastery of Procedures

This theme captures the experience of being skillful and capable in performing various procedures relevant to vocational rehabilitation. Participants expressed a sense of agency through influencing and motivating the patient and exerting influence in the decision-making of other actors. To define a reasonable goal based on the patient’s ability and wishes, followed by continual evaluation, was expressed as an important part of the motivational work. Furthermore, helping the patient to take the next step in the process by giving reasons and explanations of why previous efforts had failed was given as examples of motivational interventions.

There is a fear of failure (…) they think about all 15 previous trials. And it is very important also to know what has been done previously (…). Many times they have done some work training with insufficient support or they increased their working hours too quickly. (Participant 7).

The experience of confidence in influencing other authorities in their decision-making in the vocational rehabilitation process was prominent in narratives of agency. Taking an active role in coordination meetings organized by the SIA was also important to master.

I try to set the tone and the agenda on those meetings. So I usually start with a small long monologue and talk a little about the process and the results so far. I also describe the patient’s current state, and motivation, and my own thoughts. My strategy has been to have a consultation with the patient some half-hour before the actual meeting (…) we have then come to an agreement about what we want to present and achieve. (Participant 6).

The management of vocational rehabilitation processes was described as implying an initiation of the process, facilitating the creation of a plan for the process, bringing the process forward and carrying out a continual evaluation.

The role is, I think, to follow up the process and support the patient and bring in the factors that are needed but also the required efforts at the clinic. During this period it is we as doctors who many times follow up the whole process, but then yes … people of other professional categories sometimes do some interventions and follow up a little further along and at times a little less. And it is important that municipal actors, like the employer, the SIA and the Employment Office, collaborate. (Participant 7).

## Discussion

The present qualitative study is the first study to investigate psychiatrists’ experience of vocational rehabilitation within psychiatry. The psychiatrists who took part in the study experienced agency as well as frustration in their role. Frustration was characterised by shortcomings in terms of expertise, support, and power. The participants reported a lack of knowledge of routines, laws, regulations, concrete methods, and relevant research into vocational rehabilitation. The participants also expressed a lack of support from the psychiatric team, and stated that other professionals, such as occupational therapists and social workers, were not always available due to work commitments. Lack of power was experienced in dealings with external agencies, such as the SIA or PES. There was a high degree of frustration with regard to the questioning of the psychiatrist’s role in vocational rehabilitation. Experience of agency was characterised by orientation, receiving sufficient support, and mastering relevant procedures.

The reported lack of expertise has also been reported in previous studies in other clinical settings. Physicians have reported a need for more knowledge about the labour market and the social security system [26, 27] and how to perform work assessments [28–30]. Difficulty balancing the roles of medical expert and treating physician has been highlighted in a number of previous studies [26, 31–33]. Establishing a working alliance, with agreement on a common goal and the tasks necessary to achieve that goal, as well as a bond between patient and counsellor, contribute to predicting the employment prospects of patients with severe mental illness [34]. A working alliance helps the patient gain autonomous motivation and become engaged in the process, and it raises their expectation of returning to work [35]. Knowledge and skills in various forms could be even more important for psychiatrists as they work with a patient group that requires more time and effort if they are to return to work [21–24]. A partial explanation for the lack of knowledge among the psychiatrists in this particular study could be that many of them had completed their specialist training in other European countries. As a result, they were not in a position to access and develop the normal means of orientation in the Swedish social security system.

Communication difficulties and limited collaboration with other agencies, such as insurance boards, have been reported in other studies [18, 19, 26, 30, 34]. The participants in this study felt that the lack of resources at the SIA and PES made collaboration difficult, and this reduced their ability to work with vocational rehabilitation. This is confirmed by the decrease in the number of reconciliation meetings with the SIA, which fell by half between 2015 and 2019 [35]. Research shows that finding suitable employment and helping a patient continue their employment is more effective than traditional vocational rehabilitation [6–8]. In Sweden, these evidence-based methods are provided by agencies outside the psychiatric sector and this division of responsibility was considered problematic.

All participants reported that rehabilitation coordinators help discharge tasks related to vocational rehabilitation. This is in line with previous research into physicians’ experiences when working with rehabilitation coordinators. In a survey conducted among a very large population of physicians who had access to rehabilitation coordinators, 60% stated that the involvement of rehabilitation coordinators was of considerable value in terms of improved quality when working with sickness certification, and 31% stated it was of moderate value [5]. In primary care, physicians have begun to notice that collaboration with other professionals was more effective following the introduction of rehabilitation coordinators [31]. On the other hand, one review [36] indicated that rehabilitation coordinators could be counterproductive in vocational rehabilitation, as their involvement is time-consuming and does not increase the return to work rate.

### Implications

In an attempt to overcome these difficulties and work towards improving the potential for psychiatrists to orientate themselves, receive sufficient support, and master relevant procedures, there is potential to make structural improvements. There is a need for education and training of psychiatrists in the vocational rehabilitation process, along with relevant research and a clear definition of the roles of the psychiatrist and insurance medicine. Case conferences and coaching by colleagues with greater expertise in rehabilitation could be further ways to improve orientation. The results suggest that the need for support could be even more pronounced for psychiatrists who completed their specialist training in countries other than Sweden.

As regards the provision of evidence-based methods to facilitate return to work, psychiatry requires better structures when collaborating with the SIA, PES, and external bodies. A coordination association is an example of an external body with the potential to raise the level of collaboration. A national report suggests that interventions by coordination associations help narrow the gap between the individual and the labour market [37].

The results indicate a need for the psychiatric team to focus more on vocational rehabilitation by bringing this issue to the fore when planning further patient interventions. There is also a need for clarification of professional roles and responsibilities within the team.

### Strengths and Limitations

This is the first qualitative study of psychiatrists’ experiences of vocational rehabilitation. It includes a relevant range of topics and a diverse participant base in terms of years of working as a psychiatrist, those who qualified in Sweden and those who qualified abroad, and representation across a variety of outpatient clinics. This has enabled a rich body of data to be collected that reflects a wide range of perspectives. The present study could provide valuable information to further develop vocational rehabilitation within psychiatry.

The number of participants in the study (n = 10) was limited and they were all based in Gothenburg. There could be local variations in the relationship between the psychiatric profession and other relevant bodies, such as the SIA and PES. Despite possible local differences within Swedish psychiatry and within the social insurance systems in different European countries, we believe that the results from this study could contribute to acquiring a deeper understanding of the difficulties facing psychiatrists when working with vocational rehabilitation. It should be borne in mind that the participants in this study completed their specialist training before insurance medicine was included in the specialist training syllabus in 2015, and these results may not be as valid in the case of psychiatrists who completed their training after 2015.

Future research dealing with further perspectives on vocational rehabilitation within the healthcare system, such as those of patients and other professionals, including rehabilitation coordinators, could contribute to a broader understanding of vocational rehabilitation.
